# The effect of *Three-Circle Post Standing (Zhanzhuang)* Qigong on the physical and psychological well-being of college students: Study protocol for a randomized controlled trial

**DOI:** 10.1097/MD.0000000000012323

**Published:** 2018-09-21

**Authors:** Yu Guo, Mingmin Xu, Jialei Zhang, Qingchuan Hu, Zhengkun Zhou, Zeren Wei, Jian Yan, Yue Chen, Jianxuan Lyu, Xiaoqian Shao, Ying Wang, Jiamei Guo, Yulong Wei

**Affiliations:** a School of Acupuncture-Moxibustion and Tuina, Beijing University of Chinese Medicine, Beijing; b Ovation Health Science and Technology Co. Ltd, ENN Group, Langfang; c School of Acupuncture-Moxibustion and Tuina, Chengdu University of Traditional Chinese Medicine, Chengdu; d International Liaison Department, World Federation of Chinese Medicine Societies, Beijing, China.

**Keywords:** college students, physical and psychological health, *Qigong state* of oneness with 3 adjustments, randomized controlled trial, T*hree-Circle Post Standing Qigong*

## Abstract

**Background:**

The physical and mental health of college students tends to continuously decline around the world due to lifestyle or behavior habits changes, and pervasive presence of the Internet. Thus it is urgent to improve their health in college life. As a traditional Qigong form is practiced mainly in a standing posture. *Three-Circle Post Standing Qigong* (TCPSQ) is suitable for regular practicing and has beneficial effects on improving the physiological function and psychological emotion by adjusting body, breathing, and mind. The aim of the 3 adjustments is to achieve a state of harmonious unity-integrating these adjustments into “one.” In this study protocol article, we will systematically explore the effectiveness and safety, feasibility of TCPSQ on physical and psychological outcomes of the college students and deeply understand the state of harmonious unity-integrating adjustments of body, breath, and mind into “one.”

**Method/design:**

We will conduct a randomized, assessor, and statistician blinded, parallel-controlled trial comparing the beneficial effect of TCPSQ in college students. A total of 80 eligible college students from the Beijing University of Chinese Medicine (BUCM) will be recruited and randomly allocated into TCPSQ training or unaltered lifestyle control group according 1:1 allocation ratio with allocation concealment. TCPSQ intervention will last 10 weeks. The study period is 18 weeks including a 10-week supervised intervention and a 8-week follow-up. The relevant physical and psychological outcomes, adverse events, and safety will be evaluated at baseline, 6 weeks (at the mid-point of intervention), 11 weeks (at the end of intervention), and 19 weeks (after the 8-week follow-up period) by blinded independent outcome assessors.

**Discussion:**

This is the first randomized controlled trial protocol from the perspective of Qigong connotation to systematically investigate the effect of *TCPSQ* for the physical and mental health of a college student population. If the results in our study prove a significant intervention effect, this would provide preliminary higher-quality evidence and establish an optimal guidance for the application of *TCPSQ* exercise program among a college student population.

**Ethics and dissemination:**

The study was approved by the ethics committee of the Beijing University of Chinese Medicine (approval number: 2018BZHYLL0109). A SPIRIT checklist is available for this protocol. The trial was registered in Chinese Clinical Trial Registry (WHO ICTRP member). Registration number: ChiCTR-BON-17010840.

## 1 Background

The World Health Organization (WHO) proposes that^[1]^ health which its definition implies a comprehensive and integrative understanding of people that includes many interrelated social, psychological, and physical factors. Young adults in college life usually involves many rapid changes in the body, mind, and social relationships, the lifestyle, and behaviors that an individual develops during this stage may remain into adulthood and impact future health status.^[2,3]^ With the progress of science and technology, the integration of digital technology within daily life is becoming the cultural norm. More college students spend a majority of their spare time in the digital devices and sedentary lifestyle becomes prevalent among college students globally.^[4]^ Research has shown a substantial decrease in levels of physical activity (PA) among college students during the transition from adolescence into adulthood around the world due to lifestyle or behavior habits changes, and pervasive presence of the Internet.^[5–8]^ It is acknowledged that physical inactivity has been considered to have independent association with the increased risk of weight gain, metabolic syndrome, hypertension, diabetes, heart disease, and even all-cause mortality.^[9–14]^As a result, college students may be a population at increased risk and susceptible to serious chronic diseases later in life.^[15,16]^ Similarly, as a result of risky behaviors and multiple stressors, mental health problems in college students are increasing steeply and becoming a worldwide public health burden.^[17–20]^ Recent national data from the Behavioral Risk Factor Surveillance System (BRFSS) indicate that^[20–23]^ various forms of psychological distresses are more frequently present among college students when compared with their age group in the general population. If left ignored and untreated, mental ill-health issues for the university student population can lead to negative outcomes such as poor academic performance, dropping out or failing out of college, engaging in other risky, dangerous behavior or antisocial behavior, physical illness, and attempting or committing suicide.^[24,25]^ However, It is surprising that only a minority of college students with mental health problems seek and receive adequate help.^[26,27]^ It is therefore urgent to cope with the stressors associated with the college environment and promote both their physical and mental health. Increasing evidences showing the relationship between PA and health have supported the viewpoint that regular exercise or PA has positive effects on physical and psychological health outcomes.^[28–31]^ Recent studies showed that exercise for 8 to 12 weeks was effective for promoting physical fitness and mental health, and improving body composition in college students.^[32–34]^ In addition, PA in adolescents improves brain function and cognitive performance and may help reduce body-mind stress and mood alterations, reductions that can improve academic performance in school.^[35–39]^ As a traditional Chinese mind-body aerobic exercise, Qigong has a thousands of years history and is based on Taoist philosophy and traditional Chinese medicine (TCM) theories.^[40–43]^

Which is a combination of postures relaxation, breathing regulation, meditation, concentration, and gentle movements designed to improve holistic health and to facilitate mind-body integration.^[41–45]^ Specifically, the word “Qigong” involves 2 theories: “Qi,” the vital energy of the body, and “Gong,” the training or cultivation of Qi.^[46,47]^ Qigong challenges the foundations of modern Western biomedical thought, sharing as it does the Eastern philosophy, allows the exerciser to strengthen and gain control over Qi, the life energy that flows in channels (meridians) in the body.^[48–51]^ Qigong exercise can be practiced as a “static” (sitting, lying, or standing) or “dynamic” (moving) style,^[52]^ its aims to achieve a harmonious flow of vital energy (Qi), blood, and fluid throughout the body by long-term practicing to relieve pathological stagnation and regulate the functional activities of meridians and visceral organs through regulated breathing, mindful concentration, and gentle movements.^[41,44,47,50,51,53–61]^ With regular practice and rehearsal of the structured postures or movements, as well as concentration on mind and breath, practitioners can achieve an efficiency of “body relaxation and mind calm” and *Tian Ren He Yi* (the theory that mankind is an integral part of nature) so as to experience mood stabilization and improved strength and fitness.^[53–62]^ Due to its significant promotion of human health and ease of learning, Qigong is appropriate for nearly anyone of any age or physical condition, especially among young people. What is more, it can be practiced any place and any time, without any special equipment.^[46,49,50,61,63,64]^ A growing research suggests that Qigong may promote beneficial changes in the central nervous system (CNS), including favorable changes in dopaminergic and other neurochemical systems as well as effective for hormonal variation in the sympathetic nervous system (SNS) and/or hypothalamic-pituitary-adrenal axis (HPAA), and glucose use in specific regions of the brain associated with mood elevation, memory, and attentional processing, including the hippocampus, prefrontal cortex, and anterior cingulate gyrus.^[41,42,46,51,56,64–74]^

*Post Standing Qigong (PSQ)*, translated as the“*Zhanzhuang Qigong,*” is a traditional Qigong form practiced mainly in a standing posture. It stresses not only body alignment, but also breathing and the mind. The word “*Post*” suggests the root of a tree, which is deep in the earth and unshakable. Though associated with *Martial Arts Qigong (MAQ)*, *PSQ* is also used for health preservation and to treat diseases.^[75]^*PSQ* can enhance Qi function through the whole exercise of body posture, breathing, and meditation to calm the brain, relax the body, and keep balance of physical and psychological; thus emphasizing on the concept of integrity is the feature of *PSQ*,^[75]^ studies reported that the functions of *PSQ* were not development of partial body functions and treatments of certain disease but comprehensive exercise by recuperating body and psychology.^[75–84]^ Simplicity and popularity are the another of feature of *PSQ*. Practitioners exercising *PSQ* do not depend on place, exercise equipment, whatever sex or different age levels^.^^[75]^ Current studies have suggested that *PSQ* training appears to have substantive benefits for adults with some physical and mental disorders such as anxiety, obsessive-compulsive symptoms, depression, insomnia, neurasthenia, angiocardiopathy, hypertension, hyperlipidemia, metabolic disturbance, digestive diseases, spinal problems, kneecap strain, osteoarthrosis, and type 2 diabetes mellitus (T2DM).^[75–84]^ For young adults, particularly the college student population, the results from few studies also indicated that *PSQ* has a potential benefit on reducing depression, stress and anxiety, building self-control, and a healthy mind, and improving attention, cognitive function, body constitution, and physical function.^[75,84–88]^

At the same time, the postures adopted by various schools differ from one another, but representative variations include the *Natural Post, Three-Circle Post, Downward Pressing Post, Rounded Post, Horse Mounting Post, Subduing Tiger Post, Shaolin Sword Finger Post, and so forth.*^[75]^*Three-Circle Post Standing Qigong (TCPSQ)* exercise is one of the common forms of *PSQ*. Standing in relaxed posture, circling the arms akin to hugging a tree trunk or holding a ball, breathing deeply and steadily, mentally focus on the rotating balls or tree trunk with a relaxed attitude, maintaining balance is central to *TCPSQ.* Compared with other *PSQ*, the advantage of *TCPSQ* is its strong symmetry, which is more significant in correcting physical imbalance and body asymmetry caused by unhealthy postures in the daily life. Moreover, it can be learned easily and is less physically and cognitively demanding compared with other *dynamic Qigong*. Which is suitable for regular practicing and has beneficial effects on improving the physiological function and psychological emotion by adjusting body, breathing, and mind.^[75]^ Several studies have indicated the beneficial effects of *TCPSQ* on reducing blood lipids, lowering blood pressure, reducing blood sugar, and glycosylated hemoglobin and have proven the benefits of *TCPSQ* on cardiopulmonary function and body morphology, additionally, significant improvement has been reported in balance, attention, cognitive function, gravitational stability, strength and flexibility, as well as pain reduction and improvement in metabolic capability, bone regeneration, physical fitness, sleep quality, psychological well-being, and immune function.^[75–77,80,83–88]^

However, few studies are randomized control trial (RCT), most have significant methodological limitations, to date, no study of high methodological quality has been conducted to investigate whether *TCPSQ* can be recommended as an effective exercise for improving the emotional state, psychological well-being and physical fitness of young adults, particularly the college student population, well-designed rigorous RCTs related to the effects of *TCPSQ* on psychological and physical well-being of college students are still scarce. In this study protocol article, we will design a RCT to systematically evaluate the effects of *TCPSQ* on physical and psychological outcomes of the college students including bioelectrical activity of cortical neurons, biomechanical balance of spinal column (dynamic and static), morphological balance of spinal column, degree of thoracic breathing and abdominal breathing, electrical activity of the heart, cardiopulmonary function, as well as self-reported symptom intensity, anxiety, depression, personality, self-esteem, quality of sleep, constitution in Chinese medicine, symptoms induced by spinal problems, and self-evaluation of Qigong training.

## 2 Method/design

### 2.1 Study objective and hypotheses

The primary aim of this RCT will be to systematically evaluate the effectiveness and safety, feasibility of *TCPSQ* on physical and psychological health of college students by observing the difference between the *TCPSQ* group and unaltered lifestyle control group.

The primary hypothesis of this study is that college students who receive a 10-week *TCPSQ* training intervention will show greater improvement in physical and psychological health than those who keep to their usual daily lifestyle, as assessed immediately after the 10-week training intervention, and these benefits will continue until the end of 8-week follow-up.

The secondary aims of this study are:

1. To investigate if *TCPSQ* is more beneficial to biomechanical balance and morphological balance of spinal in college students than usual daily lifestyle.
2. To investigate the effect of *TCPSQ* on specific domains of breathing degree and heart activity in college student.
3. To explore the mechanism of action of *TCPSQ* on psychological health in college students based on electroencephalogram and psychological self-rating scales.
4. To verify whether the gains in both physical and psychological capacities related to balance and mobility and quality of life of college students trained by *TCPSQ.*

We hypothesis that college students with subhealthy state who receive a 10-week *TCPSQ* training intervention will achieve greater improvement in specific domains of spinal balance, breathing degree, heart activity, and show favorable changes in function of related brain regions than those who maintain their usual daily lifestyle.

### 2.2 Study design

**Figure 1:**
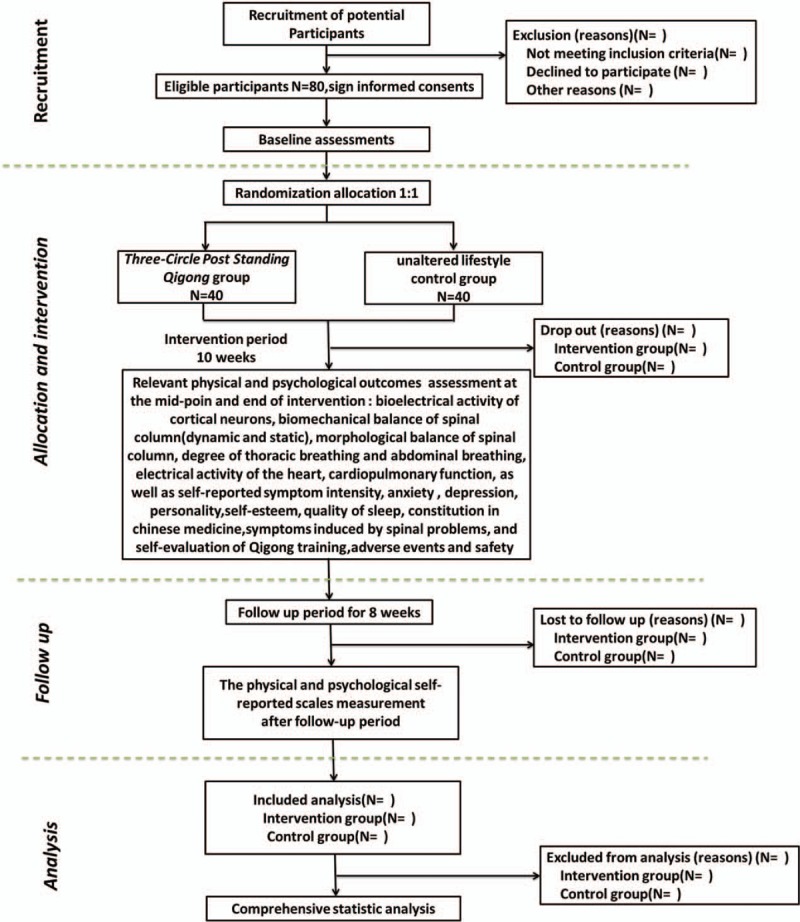
Flow diagram of study design.

This study was designed as a pilot 2-arm, randomized, assessor and statistician blinded, parallel-controlled trial comparing the beneficial effect of *TCPSQ* in college students with unaltered lifestyle control repeatedly. A total of 80 eligible college students from the Beijing University of Chinese Medicine (BUCM) will be recruited and randomly allocated to either the *TCPSQ* group or control group (unaltered lifestyle) according to 1:1 allocation ratio with allocation concealment. The participants in the *TCPSQ* group will accept a 10-week *TCPSQ* exercise training, at the same time the others in the control group will maintain their original lifestyle. The study period is 18 weeks including a 10-week supervised intervention and a 8-week follow-up with relevant physical and psychological outcomes will be measured at baseline, 6 weeks (at the mid-point of intervention), 11 weeks (at the end of intervention), and 19 weeks (after the 8-week follow-up period) by the assessors who are not participated in this trial at the Qigong and Human Science Laboratory of BUCM. The statistic analysis will be performed by special statisticians who are not involved in this trial. The flow diagram for this trial is presented in Figure 1.

### 2.3 Sample size

There are no previous clinical trials to explore the beneficial effect of *TCPSQ* on college students from the integrating with physical fitness and psychological well-being. Therefore, this study is designed as a pilot study to calculate the appropriate sample size for future randomized clinical trials. Each group should include 30 participants, which is the minimum sample size for evaluating the effect of *TCPSQ*. Considering a 20% attrition rate, a total of 80 participants are necessary, with 40 participants in each group.

### 2.4 Participant and recruitment

A total of 80 college students from BUCM who are in the first or second grade aged 18 to 25 years will be recruited into the study. Recruitment plan of participants will be performed at the campus of BUCM by schoolyard media advertisement, posting up posters, and school radio, sending leaflets and brochures. Those who are interested in taking part will contact the research assistants directly who will determine eligibility according to the inclusion and exclusion criteria at the recruitment office. The research assistants will briefly explain the purpose of the study and ask if they have an interest in participating. If an applicant meets the study criteria, he or she will be required to sign a written informed consent agreement before enrollment in this trial. Participants will also be asked to sign a declaration stating that they will participate fully to the best of their ability and will continue their involvement in the *TCPSQ* program once commenced. A log book will be given to each participant to monitor involvement and practice *TCPSQ* as well as any reporting of adverse events (AEs).

### 2.5 Inclusion criteria

Participants meeting the following criteria will be included. They must be:

(1) college students, be a fulltime student at first or second grade, be aged 18 to 25 years;
(2) right handed
(3) voluntarily participate in this research
(4) able to provide the written informed consent form for participation in the trial and take part in *TCPSQ* training on time,
(5) truthfully fill out the training record form and cooperate with the physical and psychological outcomes measure.

### 2.6 Exclusion criteria

Participants meeting the following criteria will be excluded:

(1) Being or having been engaged in a long-term regular practice of *PSQ* or other forms of *Qigong* or athletic sports;
(2) Being a member of the Martial Arts Association, Dance Association, Aerobics Association, Sanda Association, or Taekwondo Association, and so forth;
(3) Those who have family history of psychosis, neurasthenia, stress disorder, personality disturbance, mental sickness induced by taking psychoactive substances;
(4) Those who have suffered from malignant tumor, severe consumptive disease, cerebrovascular disease, communicable disease, mental illness, and severe cardiovascular, liver, kidney, gastrointestinal and hematological diseases, musculoskeletal system diseases, or other contraindication to mild- to moderate-physical exertion;
(5) Those who used antipsychotic drugs or anti-insomnia drugs at a month before the start of the study;
(6) Those who with a metal or heart pacemaker implanted in the body;
(7) Those who being or having been participated in other clinical trials that affect the physical and psychological outcomes of this study.
(8) Those who inability to comprehend and complete the study assessments or comply with the study instructions.

### 2.7 Withdrawal criteria

Participants will be withdrawn from the trial if they present any of the following conditions: poor compliance (mean compliance < 85% at the last estimation) or noncompliance, occurrence of a serious adverse event, initiative exits are unable to progress because of sudden disease, members in the control group have regularly engaged in *TCPSQ*, request to be withdrawn from the trial.

### 2.8 Randomization and allocation concealment

Randomization will be performed at post-baseline assessment and stratified by age and sex. The random allocation sequence will be generated using the PLAN sentences of Statistic Package for Social Science 21.0 (SPSS 21.0) by an independent statistician who will be not involve in this trial. The eligible participants will be allocated to either *TCPSQ* group or unaltered lifestyle control group according to 1:1 equal proportion rule. To make sure that the risk of bias remains low, participants will be registered in the database by means of a participant ID code so that assessors are blinded during the analysis. The random allocation sequence will be managed by a specified project manager who is not involved in the recruitment program of this trail, and be concealed to the screeners and outcome assessors. Randomized group assignments were sealed in opaque white envelopes. Allocation concealment will be ensured, as the service will not release the randomization code until the participants are recruited into the trial after all baseline measurements are completed. Once participants consented to enter the trial, envelopes were opened and assignments made to *TCPSQ* group or control group. The eligible participants will be informed their allocation result by the project manager via telephone after post-baseline information assessment.

### 2.9 Informed consent

Prior to the study, the general study process and the responsibilities of both participants and researchers will be explained to potential participants. They will be told that their entry into the trial is entirely voluntary and that they can withdraw at any time. In the event of their withdrawal, the data collected cannot be erased and will be used in the final analyses. Written informed consent should be obtained from each participant before any interventions related to the study are started. A research assistant will be responsible for obtaining informed consent from all participants.

### 2.10 Blinding

In this trial, it is impossible to blind the participants and Qigong exercise coaches due to this being a nonpharmacological intervention trial. Nevertheless, 2 kinds of blind code will be used to blind the outcome assessors and statistician, and we will assign a specified project manager to be in charge of the management of the random allocation sequence and blind code of the participants’ allocation result will be replaced by using alphabet “A” or “B.” And the real meaning of “A” or “B” will be signed in the second blind code. A twice unclosed blind code will be performed in this trial. Firstly, after close of data-base, the project manager will deliver the group code “A” or “B” of participants to the statistician. Secondly, the project manager will declare the real meaning of group “A” or “B” after analysis of all data is completed. Furthermore, we will rule that each investigator has a well-defined obligation: the project manager and Qigong exercise coaches will not take part in the assessment of outcome; at the same time, the outcome assessors, laboratory technicians, data managers, and the statistic analyzer will be not involved in the participants’ screening and allocating. The allocation sequence and blind code will be preserved by an independent project manager who is irrelevant with the recruitment, intervention, assessment, and statistic analysis until the statistical analysis is completed.

### 2.11 Intervention

#### 2.11.1 Three-Circle Post Standing Qigong group

**Figure 2:**
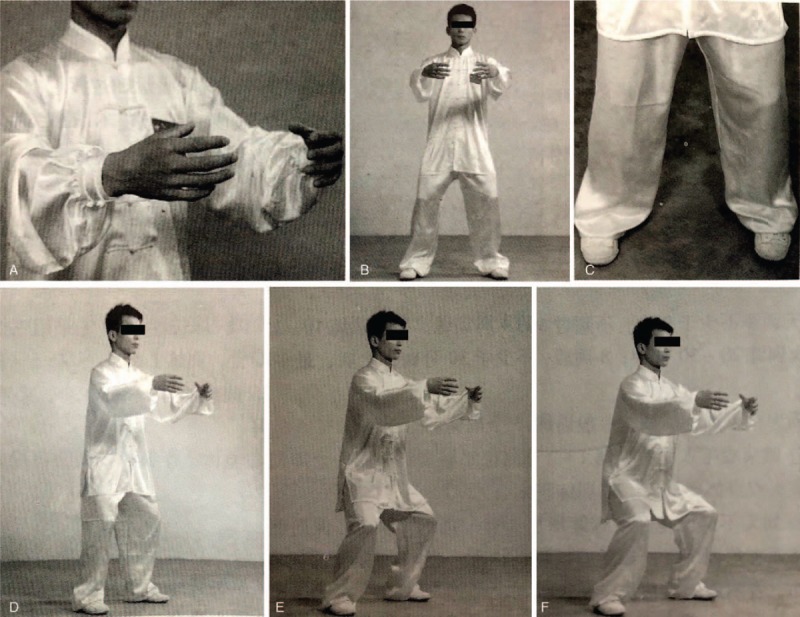
The postures of *TCPSQ*. (A) The circle of between the hands. (B) The circle of arms. (C) The circle of arches of the feet. (D) High postures of *TCPSQ*. (E) Medium postures of *TCPSQ*. (F) Medium postures of *TCPSQ*.

The *TCPSQ* training will be performed lasting 10 weeks to the participants in the *TCPSQ* group at the gymnasiums of the university. The training scheme originated from the *TCPSQ* recorded in *Traditional Chinese Medicine Qigong*^[75]^ (“13th Five-Year” planning teaching materials of China National Higher Education of Chinese Medicine, Beijing: China Press of Traditional Chinese Medicine, chief editor: Tianjin Liu, Wenchun Zhang) and *Chinese Medicine Qigong Training Guidance*^[89]^ (Beijing: China Press of Traditional Chinese Medicine, chief editor: Yulong Wei) which not only is a kind of ancient Chinese health preserving technique but also is the basis of *dynamic Qigong*. *TCPSQ* exercise characterized by interplay between obviously bilateral symmetrical physical body posture (symmetrical arches of feet present circle, symmetrical arms present circle, symmetrical hands present circle), breathing control, a meditative state of mind, and mental focus in a harmonious and relaxed manner (Fig. 2). Two qualified coaches who have engaged in the *PSQ* education at least 10 years will correct the *TCPSQ* posture during the whole intervention period.

#### 2.11.2 Control group

The participants in the control group will not receive any specific training. They will be informed to maintain their original daily lifestyle in the intervention period (based on the initial inclusion criteria that they were not exercising on a regular basis) and requested not to commence any regular exercise regime or participate in other mind-body exercises, such as yoga or other forms of Qigong. On the completion of the study they will be given the same 10 weeks of *TCPSQ* training after the 19 weeks so as to increase involvement compliance rate.

#### 2.11.3 Intervention regimen

*TCPSQ* training consisted of an initial workshop conducted over 3 consecutive half-days by 2 qualified coaches. Participants received concentrated training: this consisted of instruction in source of *PSQ*, understanding in the essence and beneficial effects of *PSQ*. Participants will be taught the relevant knowledge of *TCPSQ*, which includes the rationale that *TCPSQ* constitutes a health benefit, the action essence of *TCPSQ* the philosophy opinion of *TCPSQ*. The instruction of *TCPSQ* will be both verbal and visual. And participants will be also obtained information about procedure of this study, and the matter needing attention of individuals in the trial. Participants will be required to learn the key movements known as “regulating body, breathe, mind” and ancillary exercises with multiple repetitions of *TCPSQ* until they master it, as will be confirmed by the professional coaches. The coaches will visit each individual daily to ensure that the movements are being correctly practiced.

Once initial concentrated training was complete, participants were asked to practice supervised training. Participants of *TCPSQ* group will undergo the regular training together for the last 10 weeks at a frequency of 5 days per week (from Monday to Friday), training will be performed for 50 minutes per day, each session will include a warm-up for 10 minutes. Specified *TCPSQ* performed and refined for 30 minutes, the practice method successively as follows: body adjustment; breathing adjustment; mind adjustment, and cooling down for 10 minutes. The instructors will visit each individual to ensure that the movements are being correctly practiced. All participants will be required to fill self-made *TCPSQ* training self-evaluation scale (including self-evaluation of training difficulty, the relaxation or tensity or pain and fatigue of various parts of body, perspiration level, saliva secretion level, body sense of warmth, breathing control state, meditative state of mind, abnormal physical and mental reaction, level of coordination between body and mind during and after training) and record their daily PA information during the intervention period. The compliance of the subjects will be assessed in terms of the number of training attended and the number of training self-evaluation scale per day.

In order to exclude bias from the exceed activity of participants, all participants in both groups will be required to record PA diaries, including the type and intensity of physical activity or exercises, as well as the sedentary time and sleeping time everyday throughout this study.

### 2.12 Follow-up period

During the 8-week unsupervised follow-up period, all of the participants will return to their original lifestyles. But all participants will be required to record their daily physical activities or sport information. The forms will be returned to the researchers for following up each day by email or mail.

A weekly gathering will be organized for discussion of health-related topics and detailed follow-up.

The physical and psychological self-reported scales will be re-evaluated at the end of follow-up period. The follow-up assessment is designed to evaluate the long-term effect of *TCPSQ* on physical and psychological health of college students.

### 2.13 Outcome assessment

Outcome measurements consist of basic characteristics of information, bioelectrical activity of cortical neurons, biomechanical balance of spinal column (dynamic and static), morphological balance of spinal column, degree of thoracic breathing and abdominal breathing, electrical activity of the heart, cardiopulmonary function, as well as self-reported symptom intensity, anxiety, depression, personality, self-esteem, quality of sleep, constitution in Chinese medicine, symptoms induced by spinal problems, and self-evaluation of Qigong training. The basic characteristics of information will be collected at baseline (1–2 weeks before randomized allocation). The relevant primary and secondary outcomes will be assessed at baseline, 6 weeks (at the mid-point of intervention), 11 weeks (at the end of intervention), and the physical and psychological self-reported scales measurement will be assessed at 19 weeks (after the 8-week follow-up period). Bioelectrical activity of cortical neurons, biomechanical balance of spinal column (dynamic and static), morphological balance of spinal column, degree of thoracic breathing and abdominal breathing, electrical activity of the heart, and cardiopulmonary function will be assessed at the Qigong and Human Science Laboratory of BUCM by experienced operators who are not otherwise involved in this study. Several outcome assessors who are incharge with the physical and psychological self-reported scales measurement will investigate the physical and psychological status of the participants (such as Symptom Checklist-90 [SCL-90],^[90]^ Self-Rating Anxiety Scale [SAS],^[91]^ Self-rating Depression Scale [SDS],^[92]^ Eysenck Personality Questionnaire-revised, Short Scale for Chinese [ EPQ-RSC],^[93,94]^ Rosenberg Self-esteem Scale [RSES],^[95]^ Pittsburgh Sleep Quality Index [PSQI],^[96]^ Constitution in Chinese Medicine Questionnaire [CCMQ],^[97]^ Self-made Questionnaires of Symptoms Induced by Spinal Problems (SQSSP), Self-made Qigong Training Self-evaluation Scale [SQTSS]) at college student activity center. A summary of all measures in the trial is shown in Table 1.

**Table 1:**
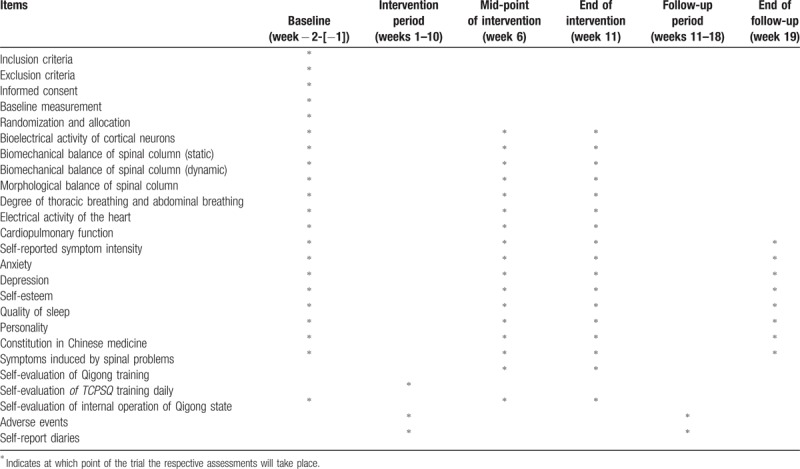
Trial measures processes chart.

#### 2.13.1 Basic characteristics variables

Demographic characteristics information of each participant's sex, age, nationality, native place, marital status, handedness, height and weight, medical history, family history of disease will be collected by the recruiters using the Self-designed Standardized Questionnaire (SSQ) at baseline. Lifestyle factors, including diet, physical activity or exercise habits, smoking habit, and drinking alcohol habit, will also be recorded. Baseline assessment will be completed before randomization.

#### 2.13.2 Primary outcomes

Primary outcomes consist of bioelectrical activity of cortical neurons, biomechanical balance of spinal column, morphological balance of spinal column, degree of thoracic breathing and abdominal breathing, electrical activity of the heart, as well as self-reported symptom intensity, anxiety, depression.

1. Bioelectrical activity of cortical neurons including electroencephalograph power spectrum of δ, θ, α1, α2, β1, β2 in *TCPSQ* state and natural stance state. It will be assessed by *the Nuamps 40 channel electroencephalograph (EEG) signal recording and analysis system and the curry-7 acquisition software* produced by Neuroscan Company, United States.
2. Biomechanical balance of spinal column including static biomechanical balance of spinal column and dynamic biomechanical balance of spinal column. Static biomechanical balance of spinal column consists of average value of foot plantar pressure, peak value of foot plantar pressure, foot contact area, center value of foot plantar pressure, center-of-gravity path, balance function index (such as vacillation range, envelope area of posturography, average barycentric coordinates, and so on) during *TCPSQ* state and natural stance state, as well as single foot and closed eye state, single foot and open eyes state. Dynamic biomechanical balance of spinal column consists of dynamic impulse in double feet, walking speed and acceleration, height change of the body's center of gravity, ground reaction force, foot pressure center track, average value of foot plantar pressure, peak value of foot plantar pressure, and foot contact area during the natural walking. It will be tested via *the Footscan Balance 7.7 acquisition and recording system and the Free STEP V.1.5.01 analysis software* produced by Sensor Medical Company, Italy.
3. Morphological balance of the spinal column consists of sagittal physiological curvature of spine, total flexion-extension activity, flexion activity, extension activity, coronal physiological curvature of spine, total left flexion-right flexion activity, left flexion activity, and right flexion activity. It will be detected with *the Spinal-mouse Spinal morphology measuring instrument* produced by Idiag, Switzerland.
4. Degree of thoracic breathing and abdominal breathing, electrical activity of the heart will be measured by *the MP150 multiple-channel electrophysiological recording device* produced by Upwards Teksystems Ltd., United States.
5. SCL-90 will be used to measure symptom intensity, which consists of the following 10 symptom factors: somatization, obsessive-compulsive disorder, interpersonal sensitivity, depression, anxiety, hostility, phobic anxiety, paranoid ideation, psychoticism, and other.^[90]^
6. Anxiety will be measured with SAS. The scale consists of 20 questions that reflect subjective feelings of anxiety symptoms. Participants are asked to respond to each question on a 4-point Likert scale ranging from 0 (never or few time) to 3 (very often or whole time). Higher composite scores indicate greater anxiety.^[91]^
7. Depression will be measured with SDS. 20 questions in SDS that reflect subjective feelings of depression symptoms. Participants are asked to respond to each question on a 4-point Likert scale ranging from 0 (never or few time) to 3 (very often or whole time). Higher composite scores indicate greater depression.^[92]^

#### 2.13.3 Testing protocol

According to new model of Qigong scientific research^[75]^ “Two-way layout, correlated detection and mutual paraphrase (2-way layout refers to design both schemes of Qigong internal operation and external detection; the whole design project includes a subjective part and an objective part. Correlated detection refers to detect objective indexes from outside and subjective operational indexes from inside simultaneously during examination. Mutual paraphrase refers to give the meaning of the experimental result by explaining internal and external indexes to each other.)” designed by Professor Liu Tianjin of BUCM. At baseline, 6 weeks (at the mid-point of intervention), 11 weeks (at the end of intervention), we designed testing scheme for *TCPSQ* internal operation (such as successively practicing feet grasping the ground (I) → stretch the waist and sink the crotch (II) → lowering the shoulders and elbows (III) → head upright and neck relaxation (IV) → the tongue tip close to the hard palate (V) → abdominal breathing (VI) → whole body relaxation (VII), and so on) and external detection (synchronous detection of bioelectrical activity of cortical neurons, static biomechanical balance of spinal column, degree of thoracic breathing and abdominal breathing, and electrical activity of the heart in *TCPSQ* state). And participants will fill in the self-made internal operation of *TCPSQ* state self-evaluation scale when the external detection is finished. At the same time, we will also performing synchronous detection of bioelectrical activity of cortical neurons, static biomechanical balance of spinal column, degree of thoracic breathing and abdominal breathing, and electrical activity of the heart in natural stance state, detection of special static biomechanical balance of spinal column (such as single foot and closed eye, single foot and open eyes), dynamic biomechanical balance of spinal column, morphological balance of spinal column, cardiopulmonary function, and the physical and psychological self-reported scales measurement. In addition, *TCPSQ* internal operations are carried out under the prerecorded instructions and it should be noted that all participants at baseline and participants in the control group just imitating internal operations under the prerecorded instructions.

#### 2.13.4 Secondary outcomes

Cardiopulmonary function, personality, self-esteem, quality of sleep, constitution in Chinese medicine, symptoms induced by spinal problems, and self-evaluation of Qigong training were regarded as secondary outcomes.

1. Cardiopulmonary function will be evaluated indirectly by blood pressure (BP), heart rate (HR). Also BP and HR will be tested by *HEM-746C electric sphygmomanometers* produced by the Omron Corp., China.
2. Personality will be measured using EPQ-RSC revised by Gong Yaoxian and Chen Zhonggeng, etc. It consists of a 48-item self-report scale with 4 subscales: an extraversion/introversion scale (lower scores indicate more introversion), a neuroticism/stability scale (higher scores indicate greater emotional instability), a psychoticism/socialization scale (higher scores indicate being more reclusive, indifferent to others, and intransigent), and a lie scale. Only the first 3 personality traits were included in our analysis. The lie scale was used to assess the acceptability of respondents’ answers. Participants provided a yes or no response, and responses are scored 1or 0, respectively, except for some reverse-scored items. These 4 traits have been shown to have good factorial similarity across 34 countries in a study that analyzed gender-specific data. In the current sample, Cronbach's coefficient for the EPQ-RSC ranged from 0.62 to 0.72.^[93,94]^
3. RSES will be adopted to measure self-esteem. We will use the Chinese version of RSES translated by Ji and Yu.^[95]^ It consists of 10 items, and the total score ranges from 10 to 40. Higher scores indicate higher self-esteem.
4. Quality of sleep will be indexed by PSQI.^[96]^ The locally validated Chinese version of the PSQI is a self-report questionnaire that assesses multiple dimensions of sleep over the past month. Nineteen individual items generate 7 component scores: subjective sleep quality, sleep latency, sleep duration, habitual sleep efficiency, sleep disturbances, use of sleeping medication, and daytime dysfunction. The sum of the 7 component scores ranges from 0 to 21; The sum of the 7 component scores yields one global score of subjective sleep quality, higher scores represent poorer subjective sleep quality.
5. Traditional Chinese Medicine body constitution will be evaluated with CCMQ. CCMQ is a self-rating scale with good reliability and validity that consists of 60 items scored on a 5-point scale, ranging from 1 (not at all) to 5 (very much). It has 9 subscales which assess one type of TCM constitution individually, including neutral constitution, Qi-deficiency constitution, Yang-deficiency constitution, Yin-deficiency constitution, Phlegm-damp constitution, Damp-heat constitution, Blood-stagnation constitution, Qi-stagnation constitution, and Special diathesis constitution. For each item, subjects selected 1 from 5 answers (not at all, few, sometimes, often, always), and given a respective score (1–5). For each subscale, the original score was first calculated by summing of the scores for each item. The derived score of each subscale was calculated from the following formula: (original score−the possible lowest score of the subscale)/(possible highest score-possible lowest score) × 100, ranging from 0 to 100 points. Neutral constitution referred to the patients with derived scores of the 8 unbalanced constitutions being <40 points, and the derived score of the Neutral constitution being ≥60 points. If the score of any constitution subscale was ≥40 points, unbalanced constitutions were diagnosed. Patients with 2 or more constitutional types ≥40 points, subjects were diagnosed with constitution of the highest subscale score.^[97,98]^
6. SQSSP will be used to evaluate subjective feelings of symptoms induced by spinal problems. It consists of 10 items that indicate 9 basic symptom factors induced by spinal problems: the relaxation or tensity or pain and fatigue of various parts of body, the degree of limitation in various parts of body, sense of suppression in the chest, palpitation, fatigue, emotional stability, insomnia, quality of life, and self-feeling in present.
7. SQTSS will be adopted to make self-evaluation about Qigong training. It has 2 subscales which assess self-evaluation during training and self-evaluation after training. The Self-made Qigong Training Self-evaluation Scale, from the perspective of participants themselves, provides a lot of information about training quality and effects of Qigong training on physiology, respiratory function, and psychology.

### 2.14 Adverse events collection procedure and safety measurements

As far as we know, AEs will be defined as any undesirable experience participants endure during the trial period, regardless of whether or not it is associated with the intervention, there are no adverse events reported about *TCPSQ*. If any unexpected AEs occur, all unexpected AEs related to *TCPSQ* will be reported to the researchers or the project manager and the causality with *TCPSQ* intervention will be analyzed regardless of the investigators’ assessments of causality and the coaches or project managers will provide the corresponding treatment to the participant. All details of AEs (defined as any functional lesion caused by the intervention, such as headache, dizziness or vertigo, distension of head, tinnitus, stuffiness in the chest and worsening shortness of breath, heart-pounding or palpitations, muscular soreness or pain, profuse cold perspiration, irritability, neurasthenia, hallucination and paranoia, psychological stress and so on), such as whether expected or not, relatedness to the study, time of occurrence, severity, management, and causality to the intervention will be recorded on Case Report Forms (CRFs) during the intervention period. All AEs will be followed-up from the date they are brought to the investigator's attention until resolution.

Serious AEs are defined that is life-threatening, requires hospitalization, results in persistent or significant disability/incapacity or death. If serious AEs happen, the researchers will report to the primary investigator and ethics committee within 24 hours, who will make a decision on whether the participant needs to withdraw from the study. The causality between serious adverse events and intervention will also be analyzed.

### 2.15 Data collection and management

The demographic and baseline characteristic data will be collected by screeners when the participants are recruited. The relevant primary and secondary outcome will be measured by the outcome assessors at baseline, 6 weeks (mid-point of intervention), 11 weeks (end of intervention), and 19 weeks (after the 8-week follow-up period). Research assistants will conduct quality control of data collection and be responsible for data entry. The project manager will be responsible for initial data cleaning, identifying, coding, and converting into the proper format for data analysis.

### 2.16 Statistical analysis

Collected data will be recorded on standardized forms. Descriptive statistics will be calculated for dependent and independent variables. This analysis will include summary statistics of demographic information and outcome measures. Continuous variables will be summarized as mean (SD) ± standard deviation for normal distribution, and median (IQR) ± interquartile range for non-normal distribution. Normality will be tested using *Kolmogorov–Smirnov* test and categorical variables will be presented with frequencies or percentages. Appropriate transformations will be applied in cases of non-normal distribution. For the variables with a normal distribution, differences in terms of outcomes between groups will be compared using an independent *T-*test. If the variables have a non-normal distribution of ordinal level, statistical comparison between groups will be made by using the *Mann– Whitney U* test even after suitable transformations. Measures with a discrete distribution will be expressed as percentages and analyzed by the *X*^*2*^ or Fisher's exact test as appropriate. Subgroup analysis will be performed according to participants’ gender, personality, and constitution of TCM. Analysis of variance (ANOVA) will be used for the repeated measurement data, and *post-hoc* comparison between groups at different assessment time points will be conducted using multiple comparisons with adjustment to the type 1 error rate. Analysis of the primary and secondary outcomes will be on the basis of intention-to-treat (ITT) population and per-protocol (PP) population. The result of the ITT analysis will be compared with that of the PP analysis to check whether the results are consistent or not. If the statistical results of the ITT and PP population data are the same, the results will be deemed to be reliable; if they are contrary, we intend to adopt the results of the ITT population. The method of processing missing data will be the last observation carried forward (LOCF). This refers to assigning the last observed value of the end point indicator to the subsequent missing evaluation point; that is, the last observation response will be considered to be the study end point. Safety will be evaluated by tabulations of AEs, and will be presented with descriptive statistics for each group. A chi-square test or a Fisher's exact test will be used to compare the frequency difference in AEs between the 2 groups, and severe AEs will be listed in detail. To explore potential factors which might influence adherence, a logistic regression model and a linear mixed-effects model will be used and adjusted for potential confounders. Adherence-related data will be taken from training log records. All statistical tests will be implemented using IBM SPSS 21.0 with bilateral inspection. The level of statistical significance is assumed at 2-sided *P* value <.05.

## 3 Ethics issue

This study protocol is conducted in accordance with the Declaration of Helsinki.^[99]^ The study protocol and consent forms were approved by the ethics committee of BUCM (approval number: 2018BZHYLL0109), where the study will take place. The researcher will explain the benefits and risks of participation in the study to each participant and will provide an informed consent form approved by the Ethics Committee. All participants will be fully informed about the trial, and will sign the voluntary written informed consent form prior to participation before the baseline assessment. All participants will have the right to withdraw from the study at any time. The research ethics committee will also be in charge of supervising all procedures carried out in our study, including participants’ recruitment, randomization, conduct of the study and data storage. In cases of changes to our study protocol, we have to hand in a written application to the research ethics committee. The committee members will then decide whether it is necessary to change the study protocol.

## 4 Dissemination

The study protocol has been registered, and is available on the Chinese Clinical Trial Register website (registered inChiCTR.org with the identifier ChiCTR-BON-17010840). This study will be published in scientific journals to target a wide range of groups if possible regardless of the magnitude or direction of effect. Study results will also be sent to study participants and disseminated to researchers, healthcare providers and healthcare professionals, as well as the general public through courses, presentations and internet regardless of the magnitude or direction of effect. No professional writers will be employed.

## 5 Discussion

The health and well-being of college or university students group is important, not only due to their potential societal influence, but also because many lifestyle-related attitudes and habits are formed at this stage and persist across the life span.^[2,3,100]^ Time at university is enjoyable for most students, but students are also exposed to various psychosocial and physical hazards, sometimes with decreased connectedness to their families.^[101]^ Qigong is one of the essential elements of traditional Chinese culture. Qigong therapy, an important branch of TCM, has a history going back thousands of year.^[40–43]^ Which is the skill of body-mind exercise that integrates the 3 adjustments of body, breath and mind into “one.”^[40–45,75]^ Compared with conventional exercise modalities (e.g., resistance training, muscular endurance training, and strength training), Qigong is simple, soft and relax, and the playing space and exercise equipment are not restrictive.^[46,49,50,61,63,64]^ In recent years, an increasing number of studies have documented the effectiveness of Qigong exercise in helping people improve their physical health and reduce anxiety, improve hedonistic tone, have beneficial effects on the cardiovascular, respiratory and endocrine systems, and strengthen the immune system.^[41,42,46,51,56,64–75,102–106]^

*PSQ* is a traditional Qigong form practiced mainly in a standing posture. During its long history, *PSQ* has branched into various styles, most of which adopt the standing posture, and hence it is known as *PSQ.* The phrase “Stand alone and guard your spirit” in *Plain Questions—On Health-Keeping of Remote Antiquity or Su Wen—Shang Gu Tian Zhen Lun* implies *post standing*. Similar phrases, such as “leaning against the wall” and “upstanding posture,” are found in *General Treatise on Etiology and Symptomology of Various Diseases or Zhu Bing Yuan Hou Lun*. *PSQ* was popularized during the 1950s as one of the main Medical Qigong forms in China. In over 60 years of clinical application, it has proven its efficacy in treating many sorts of disease.^[75]^ As we know, *PSQ* is practiced mainly in a standing posture. During practice, the upper body and 4 limbs are kept in a fixed posture to train the static force of the muscles and to keep the mind focused. On the one hand, it helps relax the central nervous system and improve the self-controlling ability of the autonomic nervous system and coordination of the peripheral nervous system. The enhancement of the overall function of the nervous system contributes to the balance and harmony within the body. On the other hand, it promotes blood circulation, improves blood supply to the organs and tissues, and increases the amount of blood flowing back to the heart, strengthening the metabolism. Thus, this form has been recognized as an important practice for strengthening the body and improving the breath, mind.^[75–88]^

*TCPSQ* is one of common forms of *PSQ*. It is characterized by 3 circles: that between the hands, the arms, and the arches of the feet, in addition to the low, medium, and high postures based on the angle of knee flexion. In terms of breath and mind adjustment, it mainly employs normal abdominal breathing and gradually increases the intensity of concentration of the mind on the lower Dantian.^[75]^ The effect of this *PSQ* on the circulatory and respiratory systems is evident. It improves respiratory efficiency and increases blood and oxygen supply in the peripheral tissues by expanding the movement of the diaphragm and regulating myptasis of the lung. Moreover, it has a comprehensive effect on the motor system, coordinating the movements of the shoulder, elbow, wrist, hip, kenn, ankle, and toe joints, and promotes return of venous blood in the lower extremities. Hence it is often used to treat arthropathy, spinal disease, microcirculatory disorders due to diabetes, and arteriolar spasm due to hypertension As an aerobic exercise, previous studies have indicated that *TCPSQ* exercise program can improve sleep quality, psychological well-being for adults.^[75–77,80,83–88]^ But there is currently a lack of evidence regarding the associations between *TCPSQ* and physical fitness, as well as self-reported symptom intensity, anxiety, depression, self-esteem, and quality of sleep. In addition, from a Qigong point of view, the content of Qigong is based on the “the 3 adjustments” of body, breath, and mind. The aim of the 3 adjustments is to achieve a state of harmonious unity-integrating these adjustments into “one.”^[40,75]^ There is a popular belief that the 3 adjustments themselves stand for Qigong. But attention needs to be paid to the rest of the definition: the state of unity. This state of oneness is the criterion that distinguishes Qigong form ordinary physical exercises, in which the 3 adjustments are practiced independently, not unified. It is believe that it is easier to achieve this state of oneness from practicing *TCPSQ.*^[75]^ However, related research is not found. In this trial, we will apply modern devices including the *Nuamps 40 channel EEG signal recording and analysis system, Footscan Balance 7.7 acquisition and recording system, Spinal-mouse Spinal morphology measuring instrument* and *MP150 multiple-channel electrophysiological recording device* to synchronously measure the change of “the 3 adjustments” of body, breath, and mind and correlation analyses state of harmonious unity-integrating these adjustments into “one” in *TCPSQ state.* Moreover, it also further compares the difference between *Qigong state* and natural state. Furthermore, we will deeply explore the influence of *TCPSQ* on college students with different personality and constitution of TCM. This trial focuses on a college student population. Through a 10-week intervention with *TCPSQ*, results from a range of primary and secondary outcome measures will provide the clear information about difference in physical and psychological outcomes between *TCPSQ* and usual unaltered lifestyle control group. In this trial, we scrupulously performed the rigorous randomized, parallel-controlled, assessor-blinded and statistician-blinded design with an appropriate sample (n = 80) to evaluate the effectiveness and safety, feasibility of *TCPSQ* on the physical and mental health of college students. We will arrange 2 qualified teachers to serve as the coaches in order to ensure the standardized intervention training for participants. Participants in *TCPSQ* group will be gathered to do the exercise at a fixed setting and time and required to fill self-made *TCPSQ* training Self-evaluation Scale. To control the trial bias, all participants in both groups will be required to record their PA and sport diary as well as the sedentary time and sleeping time everyday throughout this study. Furthermore, the result evaluators and statistical analysts will be blinded to ensure the authenticity and objectivity of the trial results.

Initially, one of strengths of this study is that strict, complete, randomization, and adequate concealment will be used in our trial. What is more, the extensive physical and psychological outcomes assessment on several levels involving bioelectrical activity of cortical neurons, biomechanical balance of spinal column morphological balance of spinal column, degree of thoracic breathing and abdominal breathing, electrical activity of the heart, cardiopulmonary function assessment will make it possible to explore the mechanism of action of *TCPSQ* which offer a comprehensive understanding on the adjustment of body, breath, and mind, allowing the researchers to investigate the relationship between subjective scales and objective indicators. At the same time, using the SQTSS to appraise training quality and effects of Qigong training on physiology, respiratory function and psychology from the perspective of participants themselves is also the unique highlight of this study.

The undeniable fact is that several potential limitations may occur in this protocol. Ideally, everyone involved in an RCT should be blinded, but this is not always feasible in the nonpharmacological trials,^[107]^ therefore performance bias may be inevitable. Although the participants and exercise coaches are not blinded and the psychological outcome measures are participants’ self-reported, the development of some form of sham intervention for use in future studies of Qigong is a desirable goal, and we will make every effort to ensure that outcome assessors, laboratory technicians, data managers, and statisticians are unaware of the treatment allocations and train the whole research team, inform the participants about the details of self-report scales. Second, all participants will come from one and the same medical university, which may decrease the sample representativeness. Third, limitations of this pilot study are the relatively small sample size (only 80 participants) because the sample size calculation process is not applied in this study. Thus we will compare the effect size between groups in order to form larger-scale trials and add subjective-objective quantitative indicators in future research.

In summary, this is the first RCT protocol from the perspective of Qigong connotation to systematically evaluate the effect of *TCPSQ* for the physical and mental health of a college student population. If this study demonstrates a significant intervention effect, this would provide a preliminary higher-quality evidence and establish a further guidance for the application of *TCPSQ* exercise program among a college student population.

## Acknowledgments

The authors thank Qigong and Human Science Laboratory of BUCM and Ovation Health Science and Technology Co. Ltd, ENN Group for equipment available for this study as well as express gratitude for all participants and researchers who participated in this trial for their cooperation. The authors also would like to deeply acknowledge Professor Tianjin Liu from BUCM and Qing Tang from Ovation Health Science and Technology Co. Ltd, ENN Group for providing valuable suggestions to conduct this study.

## Author contributions

**Conceptualization:** Yu Guo, Mingmin Xu, Yulong Wei.

**Data curation:** Jian Yan, Jialei Zhang, Yue Chen, Xiaoqian Shao.

**Formal analysis:** Ying Wang, Jiamei Guo.

**Investigation:** Yu Guo, Mingmin Xu, Zhengkun Zhou, Zeren Wei.

**Methodology:** Yu Guo, Mingmin Xu, Yulong Wei.

**Project administration:** Yu Guo, Mingmin Xu, Yulong Wei, Jialei Zhang

**Supervision:** Yu Guo, Mingmin Xu, Yulong Wei.

**Validation:** Yu Guo, Mingmin Xu, Qingchuan Hu, Jianxuan Lyu.

**Visualization:** Yu Guo, Mingmin Xu, Yulong Wei, Jialei Zhang, Qingchuan Hu, Zhengkun Zhou, Zeren Wei, Jian Yan, Yue Chen, Jianxuan Lyu, Xiaoqian Shao, Ying Wang, Jiamei Guo.

**Writing – original draft:** Yu Guo, Mingmin Xu.

**Writing – review & editing:** Yulong Wei, Qingchuan Hu, Jianxuan Lyu.

**Conceptualization:** Yu Guo, Mingmin Xu, Zhengkun Zhou, Yulong Wei.

**Data curation:** Jialei Zhang, Jian Yan, Yue Chen, Xiaoqian Shao.

**Investigation:** Yu Guo, Mingmin Xu, Zhengkun Zhou, Zeren Wei.

**Methodology:** Yu Guo, Mingmin Xu, Yulong Wei.

**Project administration:** Yu Guo, Mingmin Xu, Jialei Zhang, Yulong Wei.

**Supervision:** Yu Guo, Mingmin Xu, Yulong Wei.

**Validation:** Yu Guo, Mingmin Xu, Qingchuan Hu, Jianxuan Lyu.

**Visualization:** Yu Guo, Mingmin Xu, Jialei Zhang, Qingchuan Hu, Zhengkun Zhou, Zeren Wei, Jian Yan, Yue Chen, Jianxuan Lyu, Xiaoqian Shao, Ying Wang, Jiamei Guo, Yulong Wei.

**Writing – original draft:** Yu Guo.

**Writing – review & editing:** Mingmin Xu, Qingchuan Hu, Jianxuan Lyu, Yulong Wei.

Yu Guo orcid: 0000-0002-1752-1254
